# Bioresorbable Polymers: Advanced Materials and 4D Printing for Tissue Engineering

**DOI:** 10.3390/polym13040563

**Published:** 2021-02-13

**Authors:** Sybele Saska, Livia Pilatti, Alberto Blay, Jamil Awad Shibli

**Affiliations:** 1 M3 Health Industria e Comercio de Produtos Medicos, Odontologicos e Correlatos S.A., Jundiaí, Sao Paulo 13212-213, Brazil; sybele.saska@plenum.bio (S.S.); livia.silva@plenum.bio (L.P.); alberto.blay@plenum.bio (A.B.); 2 Department of Periodontology and Oral Implantology, Dental Research Division, University of Guarulhos, Guarulhos, Sao Paulo 07023-070, Brazil

**Keywords:** advanced polymers, 4D printing, tissue engineering, bioresorbable polymers

## Abstract

Three-dimensional (3D) printing is a valuable tool in the production of complexes structures with specific shapes for tissue engineering. Differently from native tissues, the printed structures are static and do not transform their shape in response to different environment changes. Stimuli-responsive biocompatible materials have emerged in the biomedical field due to the ability of responding to other stimuli (physical, chemical, and/or biological), resulting in microstructures modifications. Four-dimensional (4D) printing arises as a new technology that implements dynamic improvements in printed structures using smart materials (stimuli-responsive materials) and/or cells. These dynamic scaffolds enable engineered tissues to undergo morphological changes in a pre-planned way. Stimuli-responsive polymeric hydrogels are the most promising material for 4D bio-fabrication because they produce a biocompatible and bioresorbable 3D shape environment similar to the extracellular matrix and allow deposition of cells on the scaffold surface as well as in the inside. Subsequently, this review presents different bioresorbable advanced polymers and discusses its use in 4D printing for tissue engineering applications.

## 1. Introduction

Tissue engineering/regenerative medicine is an interdisciplinary area, addressing cell-based therapies and the use of bioactive and porous materials with the objective of developing functional substitutes for the repair or replacement of tissues/organs affected by an injury or disease [1]. Tissue engineering is based on three elements that must be in synergy: 1. Matrix (scaffold), 2. cells (stem cells or primary lineages), and 3. signals (mechanical, physical, electrical and/or molecules: Proteins, peptides, and cytokines) [2]. The scaffold, being a key factor, is responsible for physical and structural support for cell growth and differentiation and transport of suitable nutrients [1]. Ideally, its topography and chemical composition should be similar to the characteristics of an extracellular matrix (extracellular matrix, ECM), mimicking an extracellular environment that favors cell-material interactions [3]. The formation of tissues inside the scaffolds (three-dimensional structures) is directly influenced by the porosity rate and pore size, and these factors must be specific for the tissue regeneration. These characteristics are essential in providing an adequate supply of oxygen to promote angiogenesis [4].

Several conventional methods have been used extensively to produce porous scaffolds over the past years, such as salt/particle leaching, foaming through gas or chemical reagents, molding by solvent or by fusion, phase separation, and lyophilization [5]. However, these methods have limitations in producing three-dimensional (3D) scaffolds. This is due to their lack of control in the formation of pores and lack of interconnected pores that favor the transport of nutrients, consequently contributing to an accelerated cell growth rate inside the scaffold. Additive manufacturing is a promising alternative to produce porous 3D scaffolds that overcome these disadvantages, especially for bone substitutes.

Additive manufacturing, also known as 3D printing, comprises a series of technologies that consists of the direct generation of objects layer-by-layer through computer-aided design (CAD, computer-aided design) and/or computer-aided manufacturing (CAM) [6]. The layer-by-layer construction process have a distinct feature that allows highly complex structures to be built quickly. This technique allows the fabrication of biological structures using tissue-like materials, such as hydrogels, to be applied for tissue regeneration [7].

Even though 3D printing poses several positive properties, printed scaffolds may not promote the necessary biological responses [8]. A native tissue exhibits constant morphological changes in response to various surrounding stimuli, while the printed structures cannot actively transform after printing. Therefore, materials capable of responding to different external stimuli over time (advanced materials) have been extensively studied to be applied in tissue regeneration processes. Stimuli-responsive biomaterials can be used in the concept of four-dimensional (4D) bioprinting, in which 3D printed scaffolds are designed to transform over time according to one or more environment stimuli [9,10]. Thereupon, this review highlights the different advanced biomaterials available for 3D printing and discusses the recent advances in 4D printing for tissue regeneration.

## 2. Printable Hydrogels

The hydrogel is a hydrophilic scaffold composed of covalent and non-covalent polymeric chains bonds, providing a 3D shape environment similar to the native extra-cellular matrix (ECM) [11,12]. Its cross-linked polymers form a porous 3D structure with a high hydration level (they swell up to 99% (*w*/*w*) concerning their dry weight) without dissolving, allowing the network to retain proteins and growing factors, as well as providing an environment for gaseous and nutrients exchange, being essential for cell growth and survival [13,14,15,16,17]. Furthermore, hydrogel 3D scaffolds are beneficial for cell transplantation and tissue engineering [18,19].

The methods used for fabrication of hydrogel scaffolds include solvent casting/leaching, gas foaming/leaching, photo-lithography, electrospinning, and 3D printing [17]. Regarding the development of printable hydrogels, the most challenging approach are the physicochemical and mechanical properties, which allow the hydrogel to hold minimally adequate mechanical properties after printing and quick gelation to ensure fidelity of form of the structure to be rebuilt [11,17,18]. The printed shape maintenance depends on the hydrogel’s rheological properties, which is related to its composition (polymer and crosslinking) [17,20].

Bioresorbability and biodegradability are required to allow scaffold degradation within the implantation site during tissue regeneration [21]. Bioresorbable polymers present four degradation stages in biological systems: Hydration, strength decrease, loss of mass integrity, and solubilization via phagocytosis [22]. The degradation rate relies on polymeric nature, quantity, pH, and environment temperature [23]. The resorption of polymers is desirable for biomedical applications once they perform their function, the polymer chain tend to break into small pieces that will be reabsorbed or eliminated from the body [24,25]. Additionally, the scaffold’s gradual degradation promotes an increase in pore size, allowing a higher rate of cell proliferation and migration [26] for subsequent replacement of newly formed tissue.

**Figure 1:**
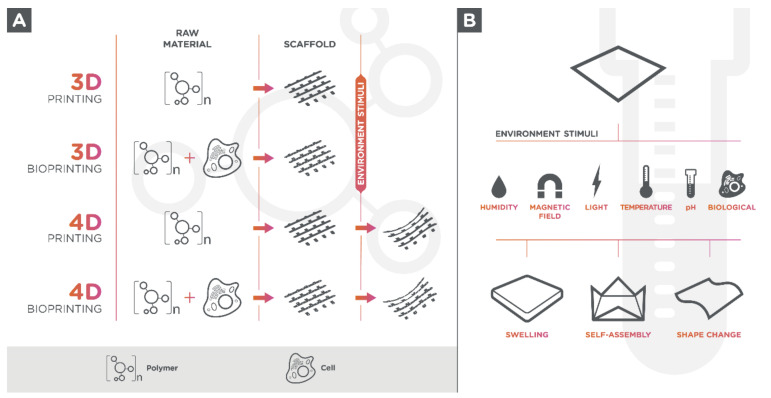
(**A**) Schematic of the different 3D or 4D printing technologies using conventional or smart materials for cell-free or cell-laden (Bioprinting) scaffolds production. Smart materials scaffolds can change their size, shape, and/or functionality in response to one or more stimulus; (**B**) types of environment stimuli and responses observed in dynamic smart materials.

Living cells can be seeded onto 3D-printed hydrogel-scaffold or can be used in a bio ink formulation since hydrogels are biologically active components for 3D printing (bioprinting) (Figure 1A) [27]. The use of tissue-specific cells in materials for 3D printing allows the creation of multifaceted and 3D-mimicked tissues, which facilitates cell adhesion due to its cell-containing products, proliferation, and differentiation once they are seeded within the structure [28,29,30].

Bioink selection for 3D bioprinting relies on several requirements, including printability, viscoelasticity, biocompatibility with living cells, tissue regeneration, resorption, shear-thinning, permeability to oxygen, nutrients, and metabolic wastes [31,32]. Furthermore, other crucial characteristics for bioinks based on hydrogels include the reversibility of gelation (relevant for pre-culture before delivery), fast gelling, and the absence of volume modification during gelling [33]. The bioink’s rheological, mechanical, and biological properties will directly impact the functionality of the final printed tissues and organs [34].

The most common 3D bioprinting techniques are the inkjet printing, microextrusion, laser-assisted printing (SLS or SLM), and stereolithography (SLA). Inkjet printing (also known as a drop-on-demand printer and direct-writing) is a fast and low-cost method in which drops of bioink liquid are ejected through thermal, electrostatic or piezoelectric actuation onto a substrate to form 3D structures in a discontinuous process [31,35,36,37]. In the microextrusion method, the bioink is extruded by a pneumatic or mechanical (piston or screw) dispensing system (needles or nozzle) in a continuous process [38,39]. In the laser-assisted printing system, a focused laser is pulsed in an absorbing layer (titanium or gold) forcing a drop of the bioink layer to deposit on substrate and form the desired structure [40,41]. The SLA technique is based on photosensitive polymers (photopolymers), acting as feedstock, which are polymerized through a UV laser light in a layer-by-layer process [42].

The biopolymers (alginate, hyaluronic acid, collagen, fibrin, fibroin, gelatin, and chitosan), are the most used polymers for the production of printable hydrogels and hydrogel-based bioinks in addition to the synthetic polymers, which include the polyethylene glycol (PEG), the Polylactide acid (PLA), the poly(lactic-co-glycolic acid) (PLGA), the polycaprolactone (PCL), and the poloxamers.

Agarose is a linear polysaccharide polymer derived from red algae, and its gelation arises through the formation of intermolecular hydrogen bonds upon cooling [43,44]. Agarose hydrogels’ viscoelastic properties depend on the source, the purification method employed, the molecular weight, and in the solution concentration [45]. These hydrogels can elicit unfavored in vivo reactions [46] and are usually used as a fugitive ink or sacrificial material in tissue engineering [47,48].

Alginate is a water-soluble polysaccharide and consists of a linear (1–4)-linked β-d-mannuronic acid (M blocks) and its C5-epimer α-l-guluronic acid (G blocks) residues [49]. The gel’s viscosity and elasticity depend on the alginate source, concentration, and the G block content [50,51,52]. It can be extracted from a brown seaweed or can be synthesized using bacterial *Pseudomonas* or *Azotobacter* [53]. This polyanionic hydrophilic polysaccharide presents a relatively short cross-linking time and is compatible with several cell types [54,55]. The hydrogel is formed when multivalent cations (usually Ca^2+^) are added to an aqueous alginate solution. Although it is mechanically unstable for a prolonged culture, alginate hydrogels present low degradation rates and cannot be used alone [56]. Alginate-based hydrogels can be applied for vascular and cartilage tissues and are extensively studied in the area of tissue engineering. [57,58,59].

Chitosan is a deacetylated form of chitin derived from shells of crustaceans [60,61]. This natural cationic polysaccharide is insoluble in water and needs to be solubilized in acid solutions [62]. The hydrogel presents relatively good mechanical stability and may be easily mixed with other hydrogels. Due to its acidity, it needs to be neutralized and can reduce cell viability. In addition, presents a limited printability due to its low mechanical strength and low gelation speed and for that reason cannot be printed alone. There are only few studies of chitosan-based hydrogels for tissue engineering [63].

Collagen, the most abundant protein in the mammalian species and marine organisms, is the primary studied natural polymer for biomaterials [64]. Collagen hydrogels are considered a suitable cell carrier that may be easily mixed with other hydrogel materials; therefore, it presents low mechanical stability and a prolonged cross-linking time (slow gelation). Likewise, it is not indicated to be used alone, and it better performs when used in polymeric composites. Type I collagen (Col-I) can self-assemble to form fibrous hydrogels at 37 °C [65]. These hydrogels have been reported in various tissue engineering applications, but mainly have been mainly utilized in cartilage and skin tissues [66,67,68].

Gelatin is a partially hydrolyzed polypeptide and is considered a form of collagen. Its gelling property depends on its source. Gelatin hydrogels present a good cell viability, a low mechanical stability, and a high solubility at a physiological temperature [69]. The thermo-responsive property functions as a cell carrier and fugitive ink, making it a good choice to be used in tissue engineering. Gelatin methacryloyl (GelMA) is a modified gelatin with a low mechanical stability [70]. Although, GelMA is compatible to many cell types, cell viability in GelMA hydrogels depends on the photocrosslinking time, which is the intensity of light and photoinitiator used to induce polymerization [71]. There are several studies of vascular, cartilage, and liver tissue engineering using gelatin and GelMA based-hydrogels [72,73,74,75,76,77,78].

The hyaluronic acid is a linear non-sulfated glycosaminoglycan (GAG) polysaccharide that requires association to other polymers as a consequence of its low mechanical stability [79]. It is commonly used to increase cell viability through cell proliferation enhancement. Due to their properties, hyaluronic acid-based hydrogels have been studied for cardiovascular and cartilage tissue engineering [80,81].

Polyethylene glycol (PEG) is the most used synthetic polymer to produce biomedical hydrogels [82]. This hydrophilic polymer can be transformed into a gel by photopolymerization [83]. PEG hydrogels present good mechanical stability and their properties may be easily manipulated using chemical modification techniques, however, they do not provide biological cues for cell proliferation [84]. The photocrosslinking time, the intensity of the light, and the photoinitiator have a great influence on cell viability. PEG-based hydrogels can be applied in different approaches to tissue regeneration, such as vascular, bone, and cartilage tissues [48,85,86,87,88].

Poly(lactic acid) (PLA) is a biocompatible synthetic hydrophobic aliphatic polyester [89] commonly used in bone tissue engineering [86,87,88,89]. The stereoisomers distribution within the polymers chains (L/D ratios) and molecular weights determines the thermal stability and the degradation properties [90].

Poly(lactic-co-glycolic acid) (PLGA) is a synthetic copolymer composed of lactic acid (LA) and glycolic acid (GA), which polymerizes through the ester linkage of their monomers [91,92]. This copolymer can be degraded by hydrolysis and the degradation time is determined by the monomer’s ratio. Considering its good mechanical strengths and structural versatility, it is often used as support structures for cartilaginous and osteochondral tissue regeneration [93,94,95,96,97]. Nonetheless, it is commonly associated with other polymers (polymeric composites) [98,99,100,101,102] since it presents poor bioactivity characteristics.

Polycaprolactone (PCL) is a thermoplastic polyester obtained by ring-opening polymerization of ε-caprolactone monomers via anionic, cationic, coordination, or radical polymerization mechanism [103]. It is a bioresorbable polymer that degrades by hydrolysis of their ester linkages. PCL may be produced with different molecular weights and shape, impacting on the degradation rate and mechanical strength [104]. PCL hydrogels present good rheological and viscoelastic properties, regulable resorption, and controllable mechanical properties; nevertheless, PCL does not have biofunctional groups to promote better surface chemistry and favor a better cell adhesion in comparison to other bioactive polymers; hence, the PCL present a low biocompatibility [105,106]. It is consequently an excellent choice of use as a supporting device, especially for hard tissues. There are several reports of its use in cardiac, bone, and cartilage engineering [106,107,108,109,110,111,112,113].

Pluronic acid (or polaxamer) is a tri-block thermoplastic copolymer consisting of a hydrophobic poly(propylene oxide) (PPO) portion and two hydrophilic poly(ethylene oxide) (PEO) portions arranged in a PEO-PPO-PEO configuration. The non-ionic surfactant gelation temperature is dependent on its concentration and structure [114]. The main characteristics of this gel form are good biocompatibility, low cytotoxicity, weak mechanical properties, quick degradation rates, rapid dissolution in aqueous solutions, and poor cell viabilities [115,116]. In the area of tissue engineering, polaxamer hydrogels have been studied for diverse approaches in tissue regeneration [114,117,118,119,120].

Biocompatible and bioresorbable polymers can also be used to produce bio-based aerogels. Aerogels are materials synthesized from gels by replacement of the solvent with a gas [121]. This replacement is carried out, after gelation step, during a supercritical fluid drying process [122]. The result is a material with a high porosity (90–99%), comprising meso and micropores (50 nm), which provides a high internal surface area and low densities [123,124,125,126,127,128]. These scaffolds can be used for tissue engineering applications due to its nanofibrous structure that are suitable for cell adhesion, proliferation and migration [129]. However the traditional technologies for aerogel production lack reproducible customization of the 3D structures and do not allow the fabrication of complex structures [121]. 3D printing of aerogel can overcome the above-mentioned shortcomings, but it requires printable sol or gel with suitable viscosity and mechanical strength. Only a few studies have been reported using 3D printing techniques [121,130,131,132,133,134,135]. Maleki et al. [135] formulated a hybrid silica–silk fibroin aerogel with an excellent printability in the wet state using a micro-extrusion based 3D printing approach. Cheng et al. [121] described a new technique that integrates direct ink writing and freeze-casting with non-toxic solvent-based inks followed by special drying techniques. Taken together, these polymers, biopolymers, or synthetic polymers, could be divided into conventional and advanced (smart) polymers according to their response to environmental [3].

## 3. Advanced Polymers

Advanced or smart materials (also called “sensitive” materials) are materials that have one or more properties or functions with the ability of responding to one or multiple external stimuli, classified as physical (temperature, humidity, electric field, magnetic field, light), chemical (pH value and ion concentration), and/or biological (enzymes and peptides) [17]. When it comes advanced polymers, these stimuli promote changes in their microstructure (Figure 1B), in which the polymeric chains can be reversibly altered in relation to hydrophilic–hydrophobic balance, conformation, solubility, or degradation [13,136]. In a vivo environment, these materials are normally responsive to multiple stimuli, and their property can be advantageous in the development of scaffolds for biomedical applications.

Temperature is the most used stimulus for biomaterials [137,138,139,140]. The hydrogels synthesis based on thermosensitive polymers has been highlighted in applications for tissue engineering, drug release, gene therapy, or biosensing, due to the sol-gel phase transition behavior of these polymers at a critical temperature [136,141]. Besides, most thermoresponsive materials enable reversible deformation [142]. Thermosensitive polymers are classified into two types regarding the critical temperature, the lower critical solution temperature (LCST), and the upper critical solution temperature (UCST). In these materials, small variations close to the critical temperature abruptly influence hydrophilic–hydrophobic interactions, often leading to a phase transition [136,143]. Therefore, UCST polymers have high solubility with an increase in temperature above its critical point. Whereas LCST are known to have a decrease in solubility when there is an increase in temperature. Recently, copolymers containing LCST close to a physiological temperature have been highlighted for the development of new materials [13], since they are good candidates for injectable and printable hydrogels for tissue engineering.

Poly(*N*-isopropylacrylamide) (PNIPAM)-based hydrogels are the most used thermoresponsive materials. These biocompatible materials are swollen in the solution at low temperature and shrink upon increase temperature above 32–35 °C (LCST). Furthermore, they present reversible folding/unfolding and may be used for 3D printing [144,145,146,147]. The main disadvantage of PNIPAM is that it is not a bioresorbable polymer [148], although there are several strategies for the development of bioresorbable PNIPAM-based hydrogels with the introduction of bioresorbable cross linkers and/or natural polymers, such as polysaccharides [149,150,151] and proteins [152], and synthetic polymers, including polyesters [153,154], PCL [138,155,156], and PEG [157,158]. Methoxy poly(ethylene glycol)-poly(pyrrolidone-co-lactide) (mPDLA, P3L7) diblock copolymer [159], poly(propylene oxide)–poly(ethylene oxide) (PEO–PPO–PEO) triblock hydrogel [160], PLGA–PEG–PLGA triblock copolymers [161,162,163] and BOX copolymer [164] are examples of bioresorbable thermoresponsive hydrogels, which not are based on PNIPAM.

Photoresponsive materials undergo physical (conformation, polarity) or chemical (hydrophilicity, charge, bond strength) transformation upon exposure to light, which can consequently result in alterations on material wettability, solubility, optical properties, and/or degradability [136]. Optical stimuli may be applied to a localized region without contact, and its dose may be easily adjusted to control response [8,165,166]. Photoresponsive systems may be divided according to light source: visible (vis), ultraviolet (UV), and near-infrared (NIR) light. UV light is powerful, yet presents low tissue penetrability and high toxicity [167]. Visible light exhibit innocuous properties and high tissue penetrability, with a weak efficiency as stimulus [168]. NIR light is an efficient stimulus for optical-responsive materials and provides low toxicity and high tissue penetration [168,169].

Optical-responsive materials present chromophores on the polymer backbone that captures the optical signal and converts the photoirradiation into a photoreaction [169]. Depending on the type of chromophore present in the reaction, it can be reversible or irreversible [170]. The most utilized photochromic compounds in polymeric systems are azobenzenes, spiropyrans, spirooxazines, diarylethenes, and fulgides [171]. Polydopamine (PDA) is a biocompatible dopamine derived from synthetic eumelanin polymer that has been widely used in biomedical engineering due to its photothermal effect [172]. Optical stimuli can also be used to induce photodegradation to certain materials [173]. One approach to tune resorption rate by light is to add photodegradable moieties (e.g., coumarinyl or o-nitrobenzyl ester) to the hydrogel [174,175].

Electric field-responsive materials are often polyelectrolyte hydrogels that can swell, shrink, erode, or bend in response to an electric field. Additionally, an electric field can be applied on cells and tissues to stimulate several biological activities such as cell adhesion and orientation, and calcium deposition [176,177,178]. The electrical stimulus is relatively easy to generate and control [179], and as a result, electro-responsive materials have been studied for several biomedical approaches, including in drug delivery [180] and cardiac tissue engineering [181]. Conductive polymers, such as poly[3,4-(ethylenedioxy)thiophene] (PEDOT), polypyrrole (PPy), and polyaniline (PANi) have been extensively studied for this purposes [182,183,184,185,186], but there are some drawbacks in using them in tissue engineering, considering their poor processability, mechanical properties and cell interaction, and lack of resorption [187,188]. To overcome these issues electroactive polymer are mixed with other polymers such as PLA, PCL, PGLA, chitosan, gelatin, and collagen [189,190,191,192]. However, even minimizing the amounts of conductive polymers, they will remain in the patient’s body [193]. Thus, bioresorbable synthesis, electrically conducting polymers (BECPs), has been a solution to overcome this issue [187]. The following hydrogels are some examples of BECPs: Gelatin-g-polyaniline/genipin [194], aniline pentamer (AP) grafting gelatin (GA) (AP-g-GA) [195], GelMA/Bio-IL, and PEGDA/Bio-IL [196].

Different polymeric materials may be functionalized with magnetic-responsive additives (micro- and nanoparticles) to respond to magnetic-field stimuli, which control the polymeric scaffold’s physical, structural, and mechanical properties [197,198]. Magnetic field-responsive materials respond and actuate according to the magnetic field’s steering in a tunable and wireless manner [199]. The type of polymer and magnetic particles, and their ratio and distribution within the matrix will determine the material response [170]. The most used additive is Fe_3_O_4_ regarding its superparamagnetic features, biocompatibility, and lack of toxicity [200,201,202,203,204,205]. PEG [203,206], polyurethane acrylate (PUA) [207], polyvinyl alcohol (PVA) [205], GelMA [208], and alginate [204] are some examples of bioresorbable polymers used in these materials.

Some materials are prompt to change their shape and size (swell or shrink) in response to humidity variation [209]. Humidity-responsive materials are composed of highly hydrophilic expandable elements and non-active rigid elements that transform the sorption or desorption of moisture into driving forces for movement [10]. These materials can occasionally return to their original state upon the removal of the stimulus (reversible). Some examples of bioresorbable polymers are cellulose [210,211], polyurethane copolymers [212], poly(ethylene glycol) diacrylate (PEGDA) [213], and PEG-conjugated azobenzene derivative (PCAD) with agarose [214].

pH-responsive polymers contain chemical groups (carboxyl, pyridine, sulfonic, phosphate, and tertiary amines) that accept or release protons in response to surrounding pH changes, resulting in structural or property changes such as solubility, degradability, conformation, activity, and self-assembly [215,216]. Once pH disparities occur in various parts of the human body, the responsiveness of these materials can be further explored in the field tissue engineering [170]. Several biocompatible and bioresorbable natural and synthetic polymers have been studied for this purpose, such as chitosan [217,218,219], hyaluronic acid [220], gelatin [221], alginic acid [222,223], dextran [224], PLGA [225], poly(histidine) (PHIS) [226], and poly(aspartic acid) (PASA) [227,228].

Other materials can be responsive to different biological stimuli, such as enzymes, oligopeptides, and proteins [229,230]. Oligopeptides and recombinant proteins have emerged as an alternative in developing smart materials, since engineering makes it possible to develop and design new polymers with a complexity and a functionality not found in nature [231]. The synthesis of recombinant natural or artificial proteins promotes the adaptation of several properties in the material including its mechanics, degradation, porosity, cell interaction, cytocompatibility, and response to external stimuli (temperature, pH, ionic forces, etc.) [15,231]. Genetically modified recombinant proteins are most likely to achieve a defined molecular structure than synthetically produced materials and may be easily modified. An example would be the fusion proteins or hybrid proteins, which consist of combining a sequence or functional domains of a specific protein with another protein sequence of interest. In such way, recombinant proteins and hybrid fusion proteins, and polynucleotides emerge as viable alternatives to produce hydrogels for three-dimensional (3D) printing.

Amongst the genetically encoded polymeric sequences, ELPs (elastin-like polypeptides), also known as elastin-like recombinamers (ELRs), have been widely used for the development of thermosensitive block copolymers for several biomedical applications, mainly for release systems and tissue engineering [232,233,234,235,236,237,238]. Elastin-like polypeptides can undergo reversible phase transition induced by pH, temperature, or ionic strength, whose transition phase is directly dependent on the ELP’s sequence transition temperature (Tt). Transition temperature varies according to the ELP’s sequence, molecular weight, and concentration. These polymers are soluble at temperatures below Tt, and insoluble at temperatures above Tt, that is, they have LCST [232]. Additionally, the mechanical properties and swelling rate of ELP hydrogels are related to the concentration, molecular weight, and content of lysine or cysteine of the monomeric sequence [239].

Cell traction forces (CTF) are the tangential tension exerted by cells on the extracellular matrix (ECM) or underlying layer, a crucial biological stimulus. The most known example is the cylindrical tubes, fabricated using traction cell force of cells seeded on a flat microplate [240]. This technique, cell origami, is based on cell traction forces by which cells transform 2D (two-dimensional) surfaces on 3D structures by folding elements in pre-defined shapes. The contractile force exerted by the cells originated from actin polymerization and actomyosin interactions, often occur in various physiological processes and are responsible for the origami folding [241]. Different scaffold properties and different mechanical forces applied on this scaffold generate various effects on the cell phenotype and metabolism [242,243,244], and it will directly impact the extracellular matrix (ECM) production, composition [245,246], and consequently, cellular traction forces [247].

Surface tension (capillary force) can also transform membranes into 3D structures [248,249,250]. An example is a capillary origami, where a liquid can be droplet on a soft film, and after the liquid evaporates, surface tension drags the fil and changes its shape [251].

In this context, smart polymers have a wide advantage in the production of hydrogels, both for bioprinting and cells carriers, or injectable drugs [252], due to their versatility and adjustability sensitivity to the stimuli of the surrounding environment, making it possible to control the desired physical-chemical and mechanical properties for each particular application.

## 4. 4D Printing in Tissue Engineering

Three-dimensional bioprinting considers only the printed object’s initial state and assumes an inanimate and static scaffold; however, placing biocompatible materials and cells through printing is not enough to construct a tissue or an organ [253]. Four-dimensional (4D) printing adds time to the process as the fourth dimension, and considers and plans changes on printed objects shapes and/or functionalities when an external or internal stimulus is imposed following the 3D printing process (Figure 1A) [142,253,254]. 4D printing is influenced by five key factors: The additive manufacturing process, the responsive material, the type of stimulus, the interaction mechanism between stimulus, and the material, and the mathematical modeling of the material transformation [170]. When exposed to appropriate stimuli, responsive materials undergo physical or chemical changes, leading to macroscopic level transformations (dimension, secondary structure, solubility, degree of intermolecular association, sol-gel transition, chain breakage) that may be useful in tissue engineering [143]. Although there are several studies using resorbable smart materials in 4D-printing for tissue engineering approaches (Table 1), scaffold degradability is a crucial property for tissue regeneration.

4D biofabrication can be performed in three ways: (i) Scaffold production, followed by material transformation and by cell seeding; (ii) scaffold production, followed by cell seeding and by material transformation; (iii) scaffold production simultaneously with cell seeding-containing material followed by material transformation [148]. However, living cells interaction with the material and/or the stimulus and/or the material transformation needs to be considered [253,275]. The materials used in the process of biofabrication must be biocompatible and non-toxic and be favorable for cell adhesion and growth. When cells are seeded prior material transformation, stimuli and transformation should not affect the viability or cell type characteristics. Considering the third approach, scaffold manufacture (bioprinting) must be suitable for cell viability. Therefore, cell traction forces depend on the cell phenotype, cell density and cell adhesion, and should be optimized for controlled conformations in printed structures [276].

In tissue engineering, vascularization is the a key factor for engineer functional tissues since it is necessary to effectively supply nutrients and oxygen, and to remove metabolic products over a distance of 100–200 mm [277,278,279]. 4D bioprinting has been intensively studied to produce blood vessel structures and microfluidic channels in different scaffolds. Cylinder-shaped structures resembling vasculature can be produced by bioprinting sacrificial hydrogels containing cells [47], or by self-folding polymers in the presence of cells [240]. Sacrificial polymers (fugitive inks), are usually applied as temporary support of overhanging structures during 3D bioprinting, and are extensively used to construct microfluidic channels which allows the creation of vascularized tissues [280]. Agarose and gelatin are the most used polymers in sacrificial strategy, where the desired channel cavity into the material is filled with these temporarily polymers during the printing process and are subsequently removed by heating [48,262,263,269]. The surfactant Pluronic F127 can be utilized as a fugitive ink for microfluidic network constructs that can also be removed through temperature [264].

There are other applications for thermoresponsive materials in tissue engineering [261,265,266,267]. Moroni et al. [268] for example, described the fabrication of 3D shape memory polymer scaffold able to change their shape in time during culture. Cells were seeded onto polyurethane/collagen type I scaffolds in a temporary shape, and during culture, due to temperature increase, the permanent shape was recovered and allowed the adherent cells to present a significantly more elongated shape. Peeters et al. [147] developed a thermoresponsive bioresorbable polymer bilayer construct (PLA-b-PEG-b-PLA/NIPAAm) that swell and subsequently roll-up under low temperatures. A catheter could deliver these cell-laden “wrap” structures to impaired myocardium, where would unroll and expose the delivered cells to the damaged tissue in response to a temperature increase to 37 °C.

Magnetic-responsive scaffolds can be applied in tissue regeneration when alignment [207], mechanical stimulation [281], and stem cell differentiation are required [282]. Magnetic field direction and strength generate specific alterations on morphology and geometry of these materials. Magnetic field-responsive materials can also be used to manipulate cell-laden printed scaffolds [276]. For this purpose, magnetic field influence on cells also needs to be considered. Stem cells loaded with magnetic nanoparticles, for example, may form 3D aggregates under magnetic fields [283].

Magnetic particles are included in the polymeric hydrogel to produce the magnetic-sensitive material. These particles may leach from the material matrix in living systems and, depending on particle size (smaller than 50 nm) and cross biological membranes, it may negatively affect the tissue functionality [284]. Therefore, the biocompatibility of magnetic particles is of extreme importance to the magnetic field-responsive materials in tissue engineering, and for this reason, iron-based particles are the most used ones [256,257]. De Santis et al. [258] analyzed the behavior of human mesenchymal stem cells (hMSCs) seeded on a 3D additive-manufactured poly(ɛ-caprolactone)/iron-doped hydroxyapatite (PCL/FeHA) nanocomposite scaffolds under a magnetic field. They demonstrated that cell adhesion and proliferation can be enhanced by employing a sinusoidal magnetic (frequency of 70 Hz and intensity of 25–30 mT). For cartilage tissue engineering, Campos et al. [259] developed an advanced bioprinting strategy incorporating magnetic field into the 3D printer with the objective of generating complex multilayers tissues with aligned collagen fiber.

Materials that are responsive to other stimuli have been developed for use in tissue engineering. Park et al. [269] controlled changes in shape of 3D printed bilayer Sil-Ma hydrogels by modulating their properties in physiological conditions through osmolarity. Based on this technique, they constructed a trachea mimetic tissue using two cell types that successfully integrated rabbit damage trachea in vivo. Another group proposed 4D bioprinting to fabricate cell-laden bilayer constructs (alginate/GelMA:alginate/PDA ) with controlled curve structures by NIR-light that can be widely used in regenerative medicine [260].

In the human body, tissues respond to small biological molecules or bio-macromolecules, such as glucose, enzymes, nucleic acids, polypeptides, and proteins [285]. Several studies focus on the development of materials associated with the responsive behavior upon being exposed to these stimuli. Burdick et al. [271] produced mimicked blood-vessel structures by seeding cells into the microchannels of a support hydrogel with RGD peptides for adhesion HUVEC cells and protease-cleavable crosslinkers for cell-mediated degradation. Once cells were exposed to angiogenic factors, the hydrogel support was degraded, and a scaffold-free cell structure was formed. Enzymes can also be utilized as biological stimuli to enhance biological activities in the scaffolds. Marquette et al. [272] entrapped two different enzymes (alkaline phosphatase and thrombin) into a 3D printed structure to attribute multiple biological activities to the scaffold. The first enzyme enabled localized and pre-programmed calcification, while the thrombin permitted the formation of fibrin biofilm.

## 5. Prospects and Conclusions

In nature, tissues are non-static functional systems able to respond to different environment changes. Although 3D printing is an indispensable tool to produce complex-shape structures for tissue engineering, the resulting printed structures are static and not capable to actively alter in response to environment variations. 4D printing has emerged recently as a technology that confers predicted dynamic transformations to printed structures in a controllable manner using responsive-materials and/or cells. However, 4D printing is still in the stage of proof-of-concept, and as it is an emerging technology, it presents several many limitations as well as challenges to overcome, such as structural design, print techniques, and ink development. There is no consistent computational model to accurately predict the material transformation over time, and technological advances are required in software and mathematical modeling [265,286]. Printing techniques using cells are recent and are constantly being improved. Achievement of higher resolution for bioprinting is always a challenge since it requires higher shear forces, negatively impacting the cell viability [148].

Responsive materials have been studied for decades but only a few have been designed for 4D printing to be applied in tissue engineering. For this purpose, these materials must be biocompatible, noncytotoxic, and preferably biodegradable (resorbable). In addition, they must present certain mechanical strength and need to be capable of performing the dynamic process in physiological environment. A crucial consideration is that the stimulus used must be safe and easy to control if applied in the presence of cells, or in the body. Extreme pH values and high temperatures, for example, should be avoided. Due to such strict requirements, only a few dynamic polymers meet all the desired qualifications. Moreover, in nature, tissues are subjected to many different stimuli and so far, most described materials are responsive to only one stimulus. Therefore, greater efforts and expertise should be applied in the development novel and multifunctional 4D inks to improve 4D printing technique.

Additionally, there are few studies on the maturation of cell-laden printed tissues, and little is known about the effect of cells on the materials shape transformation. In conclusion, 4D printing is a visionary, promising, and powerful technology mimicking the organization and biological functionality of native tissues. However, there are necessary improvements before this technology is qualified for clinical applications.

## Figures and Tables

**Table 1:** Examples of 4D-printed resorbable materials in tissue engineering.

Stimulus		Material Composition	Fabrication Method	Cells	Tissue Engineering Application	Reference
Physical	Electric field	Pluronic F127/AT-PEI	Microextrusion	No cells were tested	Muscle and cardiac and nerve tissue	[255]
	Magnetic field	Fe_3_O_4_/BP/PLA	Inkjet	No cells were tested	Cardiovascular implant	[256]
	Magnetic field	Fe_3_O_4_/MBG/PCL	Microextrusion	hBMSCs (biocompatibility)	Bone regeneration	[257]
	Magnetic field	PCL/FeHA 80/20	Microextrusion	hMSCs (seeded after printing, before stimulus)	Bone regeneration	[258]
	Magnetic field	Cell-laden Collagen/Agarose/iron	Inkjet	hKAC (bioprinting)	Cartilage regeneration	[259]
	NIR light (808 nm)	Cell-laden alginate/GelMA: alginate/PDA	Microextrusion	293T (bioprinting)	Vascularized scaffolds	[260]
	Temperature	HBC-MA	SLA	No cells were tested	Vascularized scaffolds	[261]
	Temperature	Cell-laden GelMA/Agarose	Microextrusion	MC3T3 (bioprinting)	Vascularized scaffolds	[48]
	Temperature	Cell-laden HA-MA:GE-MA	Microextrusion	HepG2/C3A (Bioprinting)	Vascularized scaffolds	[262]
	Temperature	Collagen/gelatin	Inkjet	Fibroblast (seeded after stimulus)	Vascularized scaffolds	[263]
	Temperature	Cell-laden GelMa/Pluronic F127	Inkjet	C3H/10T1/2 (bioprinting)	Vascularized scaffolds	[264]
	Temperature	SOEA	SLA	hMSCs (biocompatibility)	Biomedical scaffolds	[265]
	Temperature	Castor oil-based polymers	Microextrusion	hMSCs (biocompatibility)	Biomedical scaffolds	[266]
	Temperature	PLA-*b*-PEG-*b*-PLA/ NIPAAm	SLS	RCm and H9C2(2–1) (seeded after printing, before stimulus)	Heart Failure treatment	[147]
	Temperature	Methacrylated PCL	SLA	No cells were tested	Tracheal stent	[267]
	Temperature	PU/collagen type I	Inkjet	hMSCs (seeded after printing, before the stimulus)	Biomedical scaffolds	[268]
Physicochemical	Osmolarity	Cell-laden Sil-MA	SLS	TBSCs and Chondrocytes (Bioprinting)	Trachea tissue	[269]
	Humidity	Gel-COOH-MA/GelMA	Inkjet	HUVECs (seeded after printing, before the stimulus)	Biomedical Scaffolds	[270]
Biological	Angiogenic Growth Factors	Ad-HA or CD-HA	Inkjet	HUVECs (seeded after printing, before stimulus)	Vascularized tissues	[271]
	Enzymatic	PEG/thrombin/alkaline phosphatase	SLS	NIH-3T3 (seeded within stimulus)	Biomedical Scaffolds	[272]
Multiple	UV light and temperature	SOEA	SLS-SLA-tandem	hMSCs (after stimulus)	Cardiac Regeneration	[273]
	Light (470 nm) and electrical field	PEGDA700 + Irgacure 2959 photoinitiator	SLA	C2C12 (seeded after printing, before stimuli)	Engineering Biological machines (bio-bots)	[274]

Ad-HA: Hyaluronic acid macromer; AT-PEI: Aniline tetramer-grafted-polyethylenimine; BP: Benzophenone; CD-HA: Hyaluronic acid macromer; FeHA: Iron with hydroxyapatite; GelMA: Gelatin methacryloyl; Gel-COOH-MA: Gelatin methacryloyl with amine groups converted into carboxyl groups; HA-MA:GE-MA: Methacrylated hyaluronic acid with gelatin ethanolamide methacrylate; MBG: Mesoporous bioactive glass; NIPAAm: *N*-isopropyacrylamide; PCL: Polycaprolactone; PDA: Polydopamine; PEG: Polyethylene glycol; PEGDA700: Poly(ethylene glycol) diacrylate 700; PLA: Polylactic acid; PU: Polyurethane; Sil-MA: Synthesized from silk fibroin (SF) and glycidyl methacrylate solution (GMA); SOEA: Soybean oil epoxidized acrylate. 293T: human cell line, derived from the HEK 293; C2C12: immortalized mouse myoblast cell line; C3H/10T1/2: Mouse embryo cell line; H9C2(2–1): Rat BDIX heart myoblast; hBMSCs: Human bone marrow stem cells; hKAC: Human knee articular cells; HepG2/C3A: Human liver cancer cell line; hMSCs: Human mesenchymal stem cells; HUVECs: Human umbilical vein endothelial cells; NIH-3T3: NIH Swiss mouse embryo cells; RCm: Rat Cardiomyocytes; TBSCs: Trophoblast stem cells.

## Data Availability

No new data were created or analyzed in this study. Data sharing is not applicable to this article.
